# VCC: Vertical Feature and Circle Combined Descriptor for 3D Place Recognition

**DOI:** 10.3390/s26041185

**Published:** 2026-02-11

**Authors:** Wenguang Li, Yongxin Ma, Jiying Ren, Jinshun Ou, Jun Zhou, Panling Huang

**Affiliations:** 1 School of Mechanical Engineering, Shandong University, Jinan 250061, China; lwg_777@mail.sdu.edu.cn (W.L.); yxma@mail.sdu.edu.cn (Y.M.); shadowing@mail.sdu.edu.cn (J.R.); oujs@mail.sdu.edu.cn (J.O.); 2 Key Laboratory of High Efficiency and Clean Mechanical Manufacture, Ministry of Education, Jinan 250061, China

**Keywords:** voxelization, vertical feature, place recognition, VCC descriptor

## Abstract

Loop closure detection remains a critical challenge in LiDAR-based SLAM, particularly for achieving robust place recognition in environments with rotational and translational variations. To extract more concise environmental representations from point clouds and improve extraction efficiency, this paper proposes a novel composite descriptor—the vertical feature and circle combined (VCC) descriptor, a novel 3D local descriptor designed for efficient and rotation-invariant place recognition. The VCC descriptor captures environmental structure by extracting vertical features from voxelized point clouds and encoding them into circular arc-based histograms, ensuring robustness to viewpoint changes. Under the same hardware, experiments conducted on different datasets demonstrate that the proposed algorithm significantly improves both feature representation efficiency and loop closure recognition performance when compared with the other descriptors, completing loop closure retrieval within 30 ms, which satisfies real-time operation requirements. The results confirm that VCC provides a compact, efficient, and rotation-invariant representation suitable for LiDAR-based SLAM systems.

## 1. Introduction

Place recognition, also known as loop closure detection, is critical for SLAM (Simultaneous Localization and Mapping) and autonomous navigation systems. It identifies previously visited locations to correct accumulated drift in maps, which enables local accuracy and global consistency, making it indispensable for reliable SLAM systems [1,2]. Meanwhile, place recognition plays a vital role in determining robot positions within a given map [3,4], which prevents robots from repeated mapping and erroneous localization in previously explored environments. Additionally, for multi-robot collaborative tasks, mutual position recognition among robots is indispensable [5,6], which enables accurate relative pose estimation between adjacent robots. In complex environments with repetitive loop structures (e.g., warehouses and tunnels), precise place recognition becomes essential for constructing metrically consistent maps [7]. Consequently, loop closure detection and relocalization techniques have attracted significant research interest, yielding numerous advancements [8].

To achieve the place recognition and relocalization goals, based on the employed sensors, current methods are primarily categorized into three approaches. Vision-based loop closure detection methods leverage richer color and texture information from the environment. As demonstrated in seminal works like ORB-SLAM [9] and BoW3D [10], these approaches extensively utilize appearance-based environmental data. Furthermore, to achieve deeper environmental understanding, researchers have increasingly integrated deep learning with visual SLAM, enabling semantic-level scene interpretation [11]. However, such methods exhibit sensitivity to lighting variations and typically require extensive training datasets. In contrast, LiDAR-based loop closure detection methods demonstrate superior robustness to lighting and viewpoint variations while achieving higher precision, as evidenced by point/line feature- [12] and shape feature- [13] based SLAM systems. However, their computing efficiency performance remains susceptible to point cloud density variations. Furthermore, multimodal approaches integrate visual and LiDAR data to jointly characterize environmental features. These methods require precise sensor calibration between cameras and LiDAR [14] and incur higher computational overhead compared to the other solutions. To achieve higher precision while maintaining real-time performance, this research investigates LiDAR-based place recognition methodologies.

Although LiDAR-based place recognition methods offer significant advantages, the field still faces critical challenges, particularly regarding how to stably and accurately extract correlative features from complex environments; the inherent sparsity of point clouds combined with density variations caused by scanning distances significantly exacerbates these challenges. Moreover, how to deeply exploit environmental information to construct effective descriptors with both translational and rotational invariance remains an open research problem. Such descriptors must simultaneously satisfy efficient construction and fast matching-retrieval capability.

**Figure 1:**
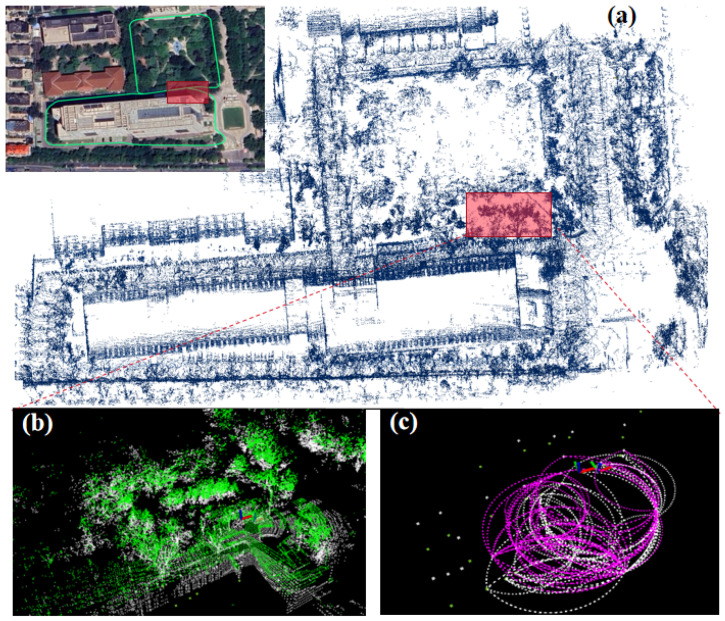
This graph encapsulates the core procedure of our method: (**a**) demonstrates the mapping results on a self-collected dataset. When the system moves to the position marked by the red box, a loop closure is successfully detected. (**b**) displays the two matched submaps involved in the loop closure: the historical point cloud is shown in white, while the current point cloud is depicted in green. (**c**) presents a subset of the descriptors corresponding to these two submaps: the historical descriptors (white color) and the current descriptors (magenta color).

In response to these challenges, this article introduces the vertical feature and circle information combined (VCC) descriptor, a novel place recognition descriptor for 3D point clouds; the core procedure of our method is shown in Figure 1.

Building upon LiDAR sensor data, the first step is to extract the inherent vertical features of the environment for descriptor construction. During this process, we mitigate the impact of point cloud density variations on feature extraction through local point cloud accumulation and voxelization. To enable the descriptor to achieve both rotational and translational invariance, we have designed a global descriptor that includes circle radius and maximum central angle. The combination of arc points endows the circular arc descriptor with viewpoint invariance. Furthermore, the types and dimensions of the vertical features that constitute the arc descriptor provide local information. The constructed circular arc descriptor enhances both the precision and robustness of place recognition. The primary contributions of this work are as follows:

- We designed one circular arc descriptor (VCC) to characterize environmental appearance, which exhibits both translational and rotational invariance while enabling 6D pose estimation.
- We propose a novel loop retrieval strategy that synergistically leverages both descriptor appearance information and their associated feature attributes, enabling stable and precise place recognition in complex, challenging environments.
- We incorporate the VCC descriptor into SLAM systems to validate its precision enhancement and error elimination capabilities. Building upon this, we further extend its application to multi-submap fusion, enabling robust integration of submaps captured from disparate viewpoints and temporal instances.

## 2. Related Works

To facilitate place recognition from sparse point clouds, descriptor-based methods are integral into the procedure. Scholars have introduced various methods aimed at extracting stable and reliable descriptors from environmental information [15,16]. The methods mainly fall into three categories: cluster-based, geometry-based, and learning-based methods.

### 2.1. Cluster-Based Methods

Among the descriptor construction process, clustering-based methods [17] are typically employed. The clustered point clouds are then assigned either artificial or natural semantic labels through geometric feature extraction [18] or knowledge distillation learning framework-based approaches [19]. To describe the environment, Gong et al. [20] segment the points cloud into multiple clusters, then the features of the clusters and the spatial relation descriptors between the clusters are extracted. In [21], one dynamic clustering strategy is proposed; for better clustering performance, an ellipse function is introduced to deal with the non-uniformity of points. To balance clustering accuracy and speed, in study [22], clustering results were firstly deduced based on angle and distance judgment, then the results were refined based on breakpoint detection. M2DP [23] and Scan-Context [24,25] encode 3D point clouds into 2D images with the purpose of utilizing image similarity methods. The NDT [26] algorithm describes a point cloud as a set of probability distribution functions and performs matching. Due to the influence of measurement angles and distances, the density of the acquired point cloud is non-uniform and it is difficult to obtain complete-object point clouds. When facing such scenarios, clustering-based methods perform poorly.

### 2.2. Geometric Property-Based Methods

On the contrary, geometric property-based methods use specific shape representations instead of point clusters from the original point cloud to realize scene reconstruction. Shapes such as circle [27], triangle [28], and linear and planner [29] are used for scene features. In study [30], one spectral method for geometric verification is proposed, then the geometric profile is re-ranked for place recognition. One hamming distance matching scheme is proposed [31], based on which TLG (TopBlock, Link, and Ground) geometric features are introduced. To extract more discriminative features, Wang [32] introduces Mercator projection principles, which convert the point cloud data into environmental depth images. In paper [33], one method based on a search box is proposed to extract pole-like features in the environment for localization purposes. One collision-model-based curb detection method and one vertical feature extraction method are proposed for intelligent vehicle self-localization [34], which are suitable for urban road scenarios. The methods employed here are predicated on specific geometric shape assumptions, which are not applicable to non-uniform scenarios.

### 2.3. Learning-Based Methods

The learning-based methods usually rely on a substantial amount of prior information to be detected or classified. Some scholars attempt to replace or add some modules of the existing network architecture to improve the detection performance. A new type of learning-based feature descriptor is generated by projecting raw data and its transformed versions into a joint binary space [35]. In another study, by applying local reference frame normalization to input data and combining it with permutation-invariant deep neural networks [36], compact descriptors with scale and rotation invariance are generated. In [37], simultaneously sampling hard positive pairs and corresponding hard negative pairs, the constructed hard training batches force the CNN model to learn more discriminative descriptors with greater effort. Li [38] provides a forward-looking perspective on the application of cross-modal LiDAR camera Transformer-based frameworks in place recognition. Xing [39] propose a segmentation network (HSSN) which combines 3D graph deep learning and laser point cloud for intelligent hand function rehabilitation. To achieve non-contact gesture recognition, ref. [40] form the hand surface point cloud recognition network (HSPCRN). Despite the deep learning methods working properly in certain situations, their effectiveness often depends on high-quality data inputs and they have poor generalization.

### 2.4. Comparative Analysis of Global Descriptors

The following Table 1 summarizes the key characteristics of state-of-the-art LiDAR descriptors compared to the proposed VCC method.

While BTC [16] and STD [28] utilize local geometric structures, the VCC descriptor introduces significant advancements in two dimensions: (1) Enhanced Semantic Saliency: BTC/STD: These methods rely on generic keypoint extraction, which can be unstable in similar or cluttered environments. VCC specifically targets vertical features (e.g., poles, pillars, and building corners). In urban and industrial settings, these are the most temporally stable landmarks. By applying vertical compression, VCC effectively filters out non-salient features (road surfaces, vegetation), leading to a higher signal-to-noise ratio in the descriptor space. (2) Higher Constraint Efficiency: BTC/STD constructing triangles from *N* keypoints results in $C_{N}^{3}$ combinations. Matching based on internal angles is highly sensitive to the precision of coordinate measurements. VCC abstracts point clusters into circular arcs. An arc not only encapsulates the distance information of a triangle but also explicitly quantifies the local curvature via the radius *R*. This higher-order geometric feature is more discriminative than simple triangle side lengths, significantly reducing the risk of perceptual aliasing.

The transition from a triangle-based representation (STD) to an arc-based representation (VCC) offers several theoretical advantages in achieving view invariance: Firstly, in a triangle, a sub-centimeter displacement of one vertex can drastically alter all three internal angles. Conversely, the circumradius *R* is a global property of the three-point set. Mathematically, *R* acts as a spatial integrator, making it more robust to the sampling noise inherent in LiDAR sensors compared to the sensitive angular metrics used in STD. Secondly, the curvature (1/R) of the arc directly corresponds to the physical spread of the environmental structure. This provides an intuitive metric for scale. For example, a set of points from a large curved wall and a set from a tight cluster of poles will yield vastly different *R* values, allowing for immediate pruning during the search process. Finally, when three points are nearly collinear, a triangle becomes “thin” and its angles become unstable. In VCC, this state is naturally handled as R→∞. This extreme value provides a clear mathematical boundary to separate linear structures from cylindrical/corner structures, a distinction that is difficult to maintain with triangle side-length ratios.

## 3. Method

**Figure 2:**
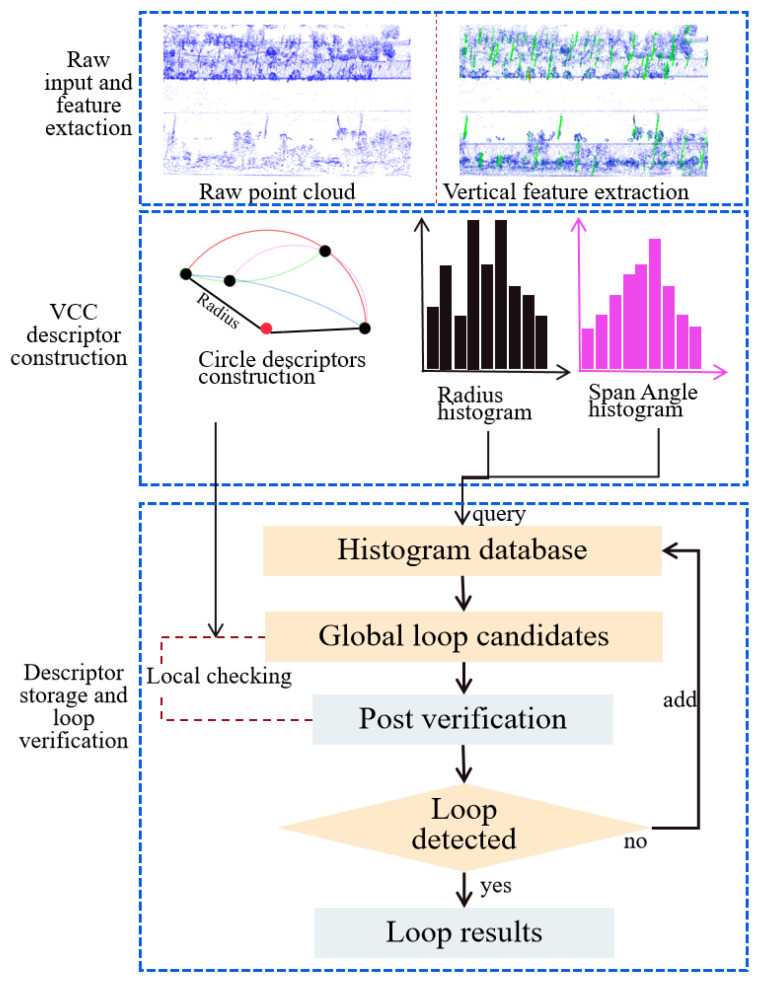
The workflow of place recognition based on the VCC descriptor. The workflow includes three main modules: vertical feature extraction, VCC descriptor construction, and loop detection based on the VCC descriptor.

As shown in Figure 2, our method consists of three main parts: (1) vertical feature extraction; (2) VCC descriptor construction; and (3) matched descriptor retrieval and relative pose estimation based on the VCC descriptor. As a result, it yields a descriptor that encodes fundamental environmental geometry, enabling effective global matching while utilizing its local attributes for false positive rejection.

### 3.1. Vertical Feature Extraction

**Figure 3:**
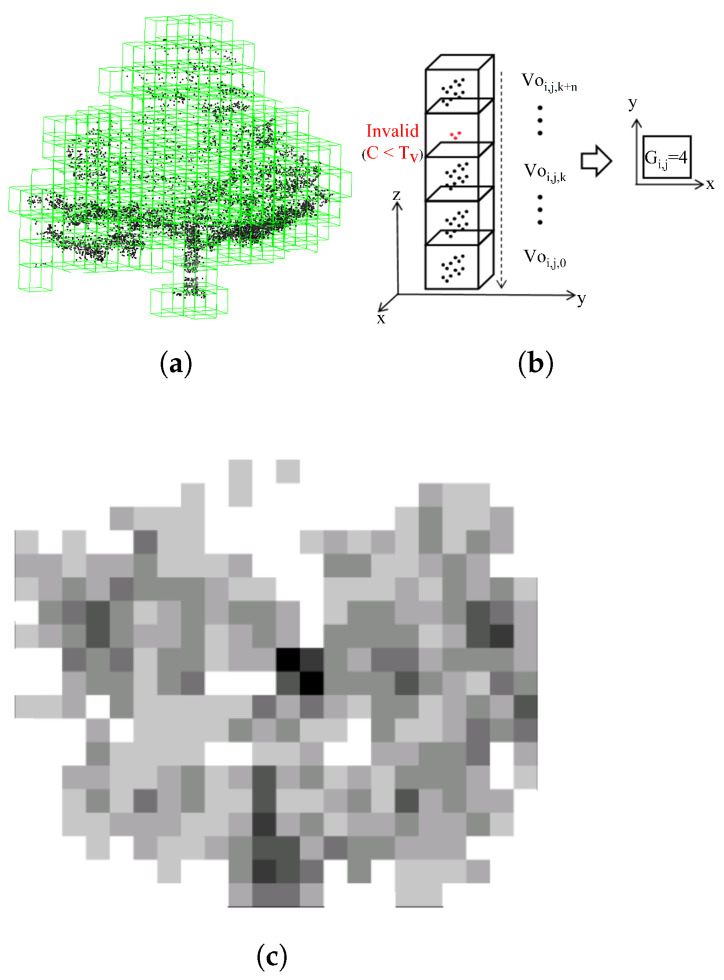
Illustration of the results for each step of dimensionality reduction analysis. (**a**) Voxel-based point cloud data. (**b**) Compression of voxel data; the voxel is invalid if the contained points number is reduced. (**c**) Generation of raster images.

To mitigate LiDAR point cloud density inhomogeneity caused by varying distances, inspired by [16], this paper adopts a multi-LiDAR scan accumulation approach, consequently reducing the number of points involved in the computation and decoupling it from cloud density. The raw point clouds are firstly segmented into voxels with the same size (Figure 3a). For the convenience of inferring feature positions, the voxel map is then compressed along the vertical direction; after this process, one grid map is obtained, with a resolution corresponding to voxel size. The value of each grid is related to the statistics of the valid voxels which lie in the corresponding row and column (Figure 3b). The calculation is denoted as Equation (1), where $G_{i,j}$ is the grid value related to the valid voxel count along the vertical direction. Based on this rule, by traversing all grid indices *i* and *j*, we obtain one grid map which describes the environmental elevation information, as shown in Figure 3c.

(1) $$G_{i,j}=\sum_{k=0}^{n}Vo_{ijk}\;\left(Vo_{ijk}=1\;if\;valid\right)$$

**Figure 4:**
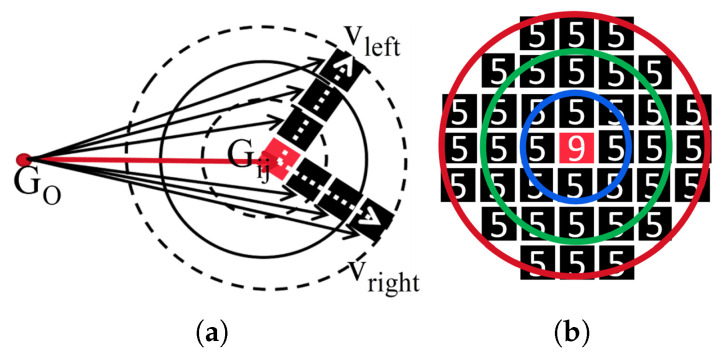
Two different types of vertical features. (**a**) Conner feature from the grids. (**b**) Isolate feature from the grids.

The grid region with a lower value refers to the areas with less ground attachments, such as roads, low shrubs, lawns, and gentle slopes. In addition, for one point cloud, the grids with smaller count values occupy the vast majority of the entire grid map. Here, we categorize these points as ground points (smaller than the ground threshold $G_{t}$). Therefore, when we exclude such grids, the computation will be significantly reduced. Among the remaining grids associated with the vertical features, we are focusing on two different characteristics. Grids with larger curvatures and grids stand out from the surroundings. Inspired by these observations, we propose one candidate region selection method based on the curvature and the local grid distribution. The candidate selection method consists of three parts: grid curvature calculation, isolation grid verification, and grid labeling. The labeled results are then used as candidates for vertical features. To calculate the curvature for each grid, we need to find the corresponding vectors $Ve_{l}$ and $Ve_{r}$ which relate to the directional vectors on the left (vectors above the red vector Figure 4a) and right sides of the grid (vectors down the red vector Figure 4a). To distinguish the direction, the angle between the grid and the neighbor grid is calculated as Equation (2). If the *z* value of vector $Ve_{ij,lm}$ is less than zero, then $Ve_{l,m}$ belongs to the left-hand element of vector $Ve_{i,j}$. By iterating through all valid grids near $G_{i,j}$, the $Ve_{l}$ and $Ve_{r}$ are obtained by Equation (3). Then we can obtain the angle $A_{i,j}$ between $Ve_{l}$ and $Ve_{r}$ by using the cosine theorem. We label the grids as corner feature areas if the angle $A_{i,j}$ falls within the interval (Amin,Amax), while those below Amin are categorized as planar features and the others larger than Amax are labeled as nonsense grids (see Table 2).

(2) $$\left\{\begin{array}{cc} Ve_{ij,lm} & =Ve_{i,j}\times Ve_{l,m}\\ Ve_{l} & =\sum Ve_{ij,lm}\;z_{Ve_{ij,lm}}<0\\ Ve_{r} & =\sum Ve_{ij,lm}\;z_{Ve_{ij,lm}}>0 \end{array}\right.$$

In Equation (2), $Ve_{l,m}$, $Ve_{i,j}$ represent the vectors between origin pose $G_{O}$ and the pose of grids $G_{l,m}$ and $G_{i,j}$. In the submap, the origin $G_{O}$ is defined at the pose of the central keyframe, which serves as the reference coordinate for the local map structure. After the above corner and planar label phase, we need to identify the isolated grid from the unlabeled grids. Equation (3) is used to obtain the relation value $R_{i,j}$ for grid $G_{i,j}$. Among Equation (3), $R_{i,j}$ represents the spatial relationship with nearby grids and $s_{r}$ represents the search radius (usually set to 3–9; the value becomes larger as the feature distribution becomes sparser). We label the grids as isolate feature areas if the $R_{i,j}$ is larger than the relation threshold $R_{t}$, as in Equation (3), and the large value of $R_{t}$ means that the grid stands out from the surrounding grids (Figure 4b).

After filtering through the above steps, the grids that are labeled as corner or isolate features are treated as the candidate grids. Such vertical features that can be distinguished are generally individual or isolate, and their predominant direction is parallel to the Z-axis.

(3) $$\begin{array}{cc} R_{i,j} & =\sum_{l=i-s_{r}}^{i+s_{r}}\sum_{m=j-s_{r}}^{j+s_{r}}\frac{G_{i,j}-G_{l,m}}{{\left(l-i\right)}^{2}+{\left(m-j\right)}^{2}} \end{array}$$

#### Derivation of Elevation Extraction

LiDAR point clouds possess inherent sparsity and irregular sampling density. To exploit these properties, we partition the 3D space into uniform cubic voxels of side length *l*. For each voxel, we perform vertical compression via vertical voxel statistics. This operation reduces the point cloud to a set of vertical feature points $v_{i}$. Let the original point set in voxel *i* be $Q_{i}={\left\{Q_{i,j}\right\}}_{j=1}^{N_{i}}$, $Q_{i,j}=\left(x_{i},y_{i},Z_{i,j}\right)$, and the vertical statistical feature is shown in Equation (4). This formulation preserves prominent vertical discontinuities (e.g., building edges and poles) while suppressing redundant ground returns (Figure 3c).

(4) $$\begin{array}{cc} V_{i} & =\left(x_{i},y_{i},z_{total}^{i}\right)\;z_{total}^{i}=\sum_{j=1}^{N_{i}}z_{i,j} \end{array}$$

To enhance vertical saliency, we compute a local curvature measure κ for each voxel based on elevation differences with neighboring voxels, as shown in Equation (5):

(5) $$\begin{array}{cc} \kappa_{i} & =1/\left(36l^{2}\right)\sum_{k\in N_{i}}\left|\begin{array}{c} z_{total}^{i}-z_{total}^{k} \end{array}\right| \end{array}$$

where $N_{i}$ denotes the 36-connected-voxel neighborhood (can also be set as a 20- or 8-voxel neighborhood). Voxels with $\kappa_{i}>\tau_{c}$ are classified as vertical features (Figure 4b); those failing this threshold or having no neighbors are marked as isolated and removed. Qualitative evaluation on different datasets shows that this step increases recall precision.

The ground threshold $h_{g}$ is chosen based on statistical analysis of elevation distribution of the environment. Empirically, we set Equation (6):

(6) $$\begin{array}{cc} h_{g} & =\mu_{z}+\alpha \sigma_{z} \end{array}$$

where $\mu_{z}$ and $\sigma_{z}$ are the mean and standard deviation of elevations in low-curvature voxels and α is a tunable parameter (typically 1.5≤α≤2.0). This criterion effectively separates ground from elevated structures while preserving sufficient vertical features for matching.

Compared to pole-like object detection (which relies on clustering vertical points and fitting cylinders), our method transforms 3D point operations into 2D grid operations, reducing complexity from O(N) to O(M×N) where $M\times N\ll N_{3}$. Unlike plane segmentation (e.g., RANSAC-based floor/wall extraction), which may discard slender vertical elements, our voxel-based curvature filter explicitly retains them.

The information from a single sparse frame may be confused with noise. The multi-frame accumulation integrates point clouds from *T* consecutive scans into a common coordinate frame via pose estimates ${\left\{T_{t}\right\}}_{t=1}^{T}$, shown in Equation (7):

(7) $$\begin{array}{cc} P_{acc} & =\overset{T}{\underset{t=1}{⋃}}\left\{T_{t}^{-1}p_{s}^{t}\right\} \end{array}$$

where $p_{s}^{t}$ is a point in scan *t*. Mathematically, for a fixed voxel *i*, the expected number of points increases linearly with *T*, filling gaps and increasing point density on surfaces. For a fixed voxel *i*, the expected number of points increases linearly with *T*, reducing the coefficient of variation $CV=\sigma_{N}/\mu_{N}$. The coefficient of variation (CV) after accumulation becomes Equation (8):

(8) $$CV_{acc}=\sqrt{\sum_{t=1}^{T}\sigma_{i}^{2}}/\left(\sum_{t=1}^{T}\mu_{i}\right)=\sqrt{T\sigma_{i}^{2}}/\left(T\mu_{i}\right)=CV_{single}/\sqrt{T}$$

where $CV_{single}$ is the CV for a single frame. Thus, multi-frame accumulation reduces density variation by a factor of $\sqrt{T}$, leading to more uniform point distributions. This homogenization improves the stability of curvature-based vertical feature extraction, as local elevation differences become less sensitive to sampling noise. This process improves robustness, accuracy, and scene understanding while balancing computational load.

### 3.2. VCC Descriptor

In this section, we present the construction method of the VCC descriptor, which consists of the vertical descriptor and the circular arc descriptor.

**Figure 5:**
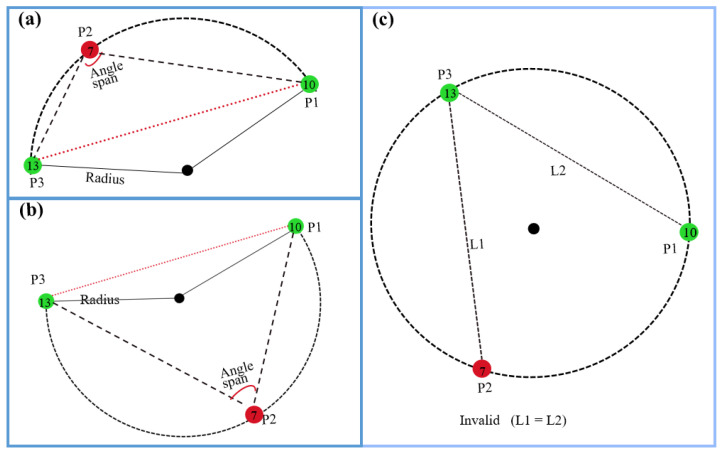
Illustration of the VCC descriptor. The red and green circles represent vertical features, where the colors indicate their categories (pole, corner), while the numbers inside indicate the vertical point cloud distribution at each location. (**a**,**b**) are valid VCC descriptors, while (**c**) is an invalid descriptor due to two similar edge lengths that create an ambiguous vertex arrangement.

(1) Vertical feature: The vertical feature is generated during point clouds projection as a grayscale value, encoding both height and categorical information of associated vertical features $V_{Des}\left\{int\;type;int\;height\right\}$. The descriptor comprises two components: (a) The feature type and height attribute characterizes the vertical distribution of point cloud data associated with the feature. (b) The 3D position of the vertical feature, which in this context is the planar coordinates of features, is determined by their 2D projection, while for vertical coordinates, we utilize the height of the nearest ground point to the feature. These 3D features provide foundational data for subsequent loop closure detection and pose estimation.

(2) Circle feature: To characterize geometric relationships among spatial points and provide comprehensive environmental representation, we introduce the circular descriptor. This design is rooted in geometric principles where three non-collinear points uniquely determine a circular arc’s radius and central angle. The triple-vertex configuration inherently ensures rigid-body rotation invariance, guaranteeing consistent radius and central angle measurements across varying viewpoints. This implicitly relies on the axiom that three spatial points define a unique circle, making circular arcs an optimal choice for characterizing spatial feature relationships. We select the closet $C_{n}$ vertical feature for the construction of the circle.

(3) VCC descriptor: The VCC descriptor integrates the strengths of both vertical and circular arc features. The VCC descriptor consists of a circular feature and three vertical features corresponding to the circle $VCC_{Des}\left\{V_{Des}:v1,v2,v3;C_{Des}:C\right\}$. This combination ensures that the circular arc descriptor maintains pose invariance of corresponding vertices, while the vertical features characterize the local distribution of point clouds, providing a basis for further refinement in descriptor matching. As illustrated in Figure 5, the VCC descriptor comprises the following elements: one circular arc descriptor, vertical feature points $\left\{P_{1},P_{2},P_{3}\right\}$, and the corresponding submap. Here, we utilize consecutive LiDAR keyframe combinations as submaps; we then extract vertical features from the submaps and construct VCC descriptors based on the criteria below.

We explicitly reject point triplets where any two edges exhibit similar lengths (see Figure 5c), as such configurations introduce vertex ordering ambiguity. Additionally, we discard circles with excessively large diameters or spanning angles. Specifically, we eliminate circles with: diameters above an upper limit $R_{max}=72$ m and spanning angles exceeding $A_{max}=$ 160°. The parameter settings are based on two key considerations: (1) excessively large-diameter circles yield poor circular arc descriptor construction due to distance effects and (2) overly wide spanning angles result in near-parallel alignment of the three vertical features, compromising the robustness of the constructed circular arc descriptors.

#### 3.2.1. Mathematical Derivation for VCC

Given three non-collinear points $P_{1}$, $P_{2}$, and $P_{3}$ in 3D space, these points uniquely define a circle with center *C*, angle span As, and radius *r*, obtained by solving Equation (9):

(9) $$\begin{array}{c} {∥P_{i}-C∥}^{2}=r^{2} \end{array}$$

Subtracting equations pairwise yields a linear system in *C*:

(10) $$\begin{array}{c} \left(P_{j}-P_{i}\right)\cdot C=\left({∥P_{j}∥}^{2}-{∥P_{i}∥}^{2}\right)/2\;i\neq j \end{array}$$

Solving this system in Equation (10) gives *C* and $r\;=\;∥P_{i}-C∥$. To eliminate directional ambiguity, we reorder the points. Firstly, we find the max side length $d_{max}=max\left\{d_{12},d_{13},d_{23}\right\}$. Then the point corresponding to the maximum side length is labeled as $P_{2}$. The two remaining points are sequentially designated as $P_{1}$ and $P_{3}$ according to their quadrant locations relative to the circle center *C*, as shown in Figure 5. The max side $d_{max}=d_{13}$. The angle span As is corresponding to this side $d_{13}$, which can be reformulated as Equation (11).

(11) $$\begin{array}{c} As=cos^{-1}\left(\left(d_{12}^{2}+d_{23}^{2}-d_{13}^{2}\right)/\left(2d_{12}d_{23}\right)\right) \end{array}$$

Under a global rotation $R_{\phi }$ and translation *t*, the projected points transform as $p_{i}^{\prime }=R_{\phi }p_{i}+t$. Substituting this into the above equations shows that $C^{\prime }=R_{\phi }C+t$. Thus, both radius and angle span remain invariant under rotation. We evaluate the stability of *r* and As under different orientations. The results show that the maximum relative error of *r* is less than 0.3% and that of As is less than 0.5%, satisfying boundary constraints for robust matching. Because the arc descriptor depends solely on intrinsic geometric relations, it is invariant under any rotation around the circle center. This property is especially beneficial in environments with frequent large-angle viewpoint changes.

Scan-Context achieves rotation invariance by cyclically shifting columns in a polar grid and selecting the best match. In contrast, our arc-based descriptor derives invariance directly from geometric constraints, avoiding exhaustive search over rotational offsets. Consequently, our method exhibits higher computational efficiency $O(N^{\prime })$ per comparison vs. for Scan-Context O(N), where $N^{\prime }$ is the circle number which is substantially lower than the cardinality of the original point set number *N*.

The data structure of the circular arc descriptor is $C_{Des}\left\{double\;r,As;Pose\;C\right\}$. The radius (r) is computed as the radius of the circle formed by three non-collinear points $\left\{P_{1},P_{2},P_{3}\right\}$. The angle span (As) corresponds to the arc subtended by the longest chord $(L_{max}=max(||P_{i}-P_{j}\left||)\right)$. To ensure consistent descriptor orientation regardless of viewpoint changes, we first identify the pair with the maximum separation distance, designated as P1 and P3. The remaining point is automatically assigned as P2. P1 and P3 are oriented in counterclockwise sequence relative to their position in the arc’s quadrant within the local coordinate system. Conversely, if two descriptors share identical radius and span angle measurements, they describe the same circular arc with corresponding vertices.

Following the geometric validation process, we construct the VCC descriptor exclusively using qualified circles that satisfy all filtering criteria for the corresponding submap. For each validated circular arc, we construct a composite descriptor comprising radius and span angle, which constitute the circular arc feature, while the vertical feature consists of the feature type and its height.

#### 3.2.2. Impact of Point Cloud Sparsity on VCC

Let $N_{v}$ denote the average number of points per voxel in the input LiDAR scan. The VCC descriptor relies on computing local curvature $\kappa_{i}$ and identifying vertical features via elevation differences between neighboring voxels. When $N_{v}$ decreases (higher sparsity), the variance in elevation estimates increases due to there being fewer samples per voxel. Assuming elevation measurements follow $N(\mu_{z},\sigma_{z}^{2}/N_{v})$, the curvature variance scales as Equation (12):

(12) $$\begin{array}{c} Var\left[\kappa_{i}\right]\propto \sigma_{z}^{2}/N_{v} \end{array}$$

**Figure 6:**
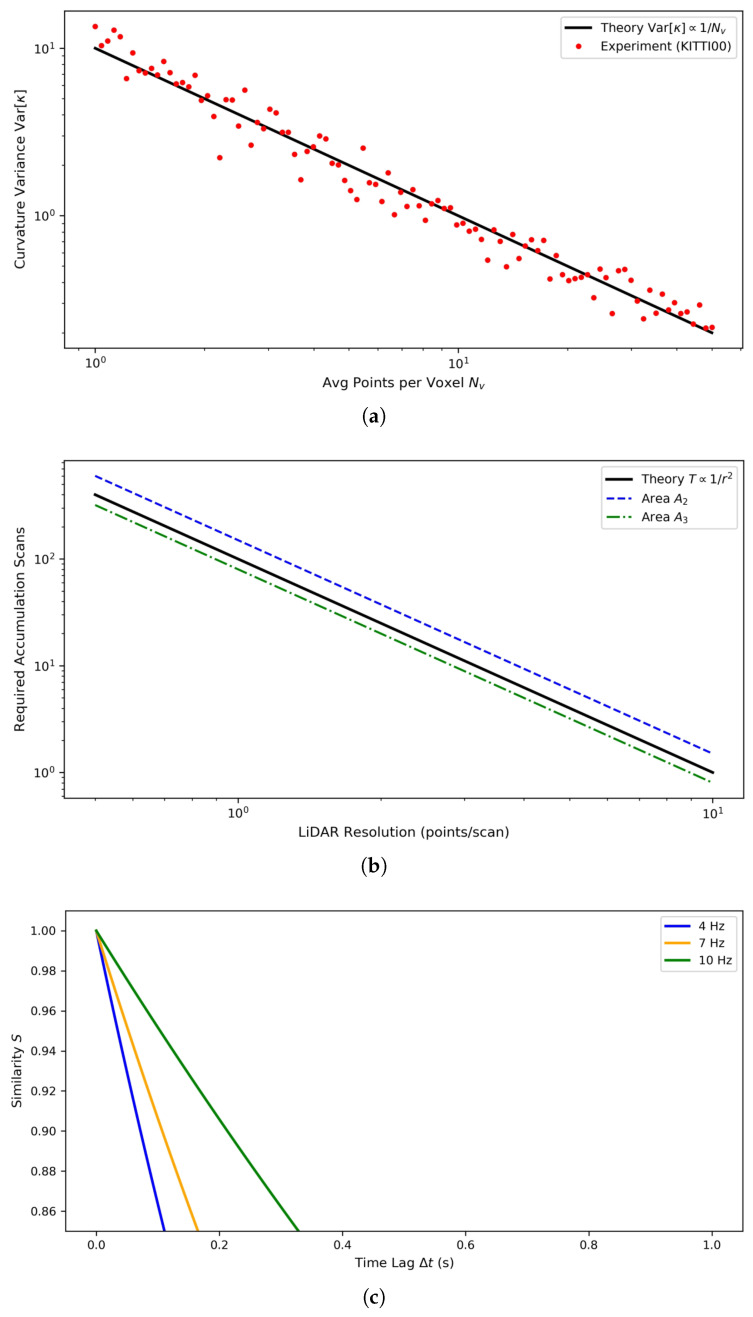
Comprehensive analysis of Vcc descriptor robustness. (**a**) illustrates the impact of sparsity on the curvature calculation of features. (**b**) depicts the relationship between point cloud sparsity and the required number of accumulation frames. (**c**) illustrates the relationship between descriptor similarity and frequency.

Empirical evaluation on the KITTI00 dataset shows that reducing $N_{v}$ from ten to two (simulating sparser scans) increases curvature variance by 4.8×, leading to a 15.3% drop in vertical feature precision Figure 6a. To mitigate this, our multi-frame accumulation strategy effectively increases $N_{v}$ by a factor of $n_{a}$, restoring descriptor stability.

Let *r* denote the LiDAR resolution (points per scan). The single-scan point density in a region of interest is proportional to $r^{2}$. For a given scene area *A*, the expected number of scans per frame is Equation (13):

(13) $$\begin{array}{c} N_{scan}\propto A\cdot r^{2} \end{array}$$

Multi-frame accumulation over $n_{a}$ frames increases the effective density to Equation (14). Thus, for a fixed $N_{eff}$, the required accumulation parameter $n_{a}$ scales inversely with $r^{2}$. This relationship indicates that low-resolution LiDARs require longer accumulation times to achieve the same descriptor stability as high-resolution systems, as shown in Figure 6b.

(14) $$\begin{array}{c} N_{eff}=n_{a}\cdot N_{scan}\propto n_{a}\cdot A\cdot r^{2}\\ n_{a}\propto 1/r^{2} \end{array}$$

To evaluate temporal robustness, we compute the descriptor similarity $S(t_{1},t_{2})$ between VCC descriptors extracted from scans at different times. We use cosine similarity in Equation (15):

(15) $$\begin{array}{c} S\left(t_{1},t_{2}\right)=d\left(t_{1}\right)\cdot d\left(t_{2}\right)/\left(∥d(t_{1})∥∥d(t_{2})∥\right) \end{array}$$

where d(t) is the VCC descriptor at time t. Experiments across scan frequencies from 4 Hz to 10 Hz show that $S(t_{1},t_{2})$ remains above 0.92 for temporal separations up to 1 s Figure 6c, indicating high temporal consistency. The slight degradation at lower frequencies is attributed to increased motion distortion between frames.

Let $z_{i,j}$ be the raw elevation measurement in voxel *i* from point *j*, $z_{i,j}=z_{i}^{true}+\eta$, contaminated by zero-mean Gaussian noise $\eta ∼N(0,\sigma^{2})$. Voxel normalization computes the mean elevation $\bar{z_{i}}=1/N_{i}\sum_{j=1}^{N_{i}}z_{i,j}$. The noise variance of the mean is Equation (16):

(16) $$\begin{array}{c} Var\left[\bar{z_{i}}\right]=\sigma^{2}/N_{i} \end{array}$$

This demonstrates that voxel normalization reduces noise by a factor of $\sqrt{N_{i}}$, with larger voxels (more points) providing greater noise suppression. For different LiDAR sensors with noise levels $\sigma_{n}^{\left(1\right)}$ and $\sigma_{n}^{\left(2\right)}$, the relative noise reduction ratio is Equation (17). This mechanism explains why VCC descriptors exhibit consistent performance across different LiDAR sensors with varying noise characteristics.

(17) $$\begin{array}{c} Var\left[{\bar{z_{i}}}^{\left(1\right)}\right]/Var\left[{\bar{z_{i}}}^{\left(2\right)}\right]=\frac{{\left(\sigma_{n}^{\left(1\right)}\right)}^{2}/N_{i}}{{\left(\sigma_{n}^{\left(2\right)}\right)}^{2}/N_{i}}={\left(\frac{\left(\sigma_{n}^{\left(1\right)}\right)}{\left(\sigma_{n}^{\left(2\right)}\right)}\right)}^{2} \end{array}$$

### 3.3. Data Maintenance

This section presents the design and implementation of our data maintenance system, specifically optimized for efficient storage and retrieval of VCC descriptors among submaps. Efficient management of dense descriptors within submaps is critical for accurate loop detection. We adopt histogram-based structures for descriptor storage and retrieval, demonstrating significant advantages over conventional data structures (k-d trees, hash tables) used in state-of-the-art systems. Following VCC descriptor extraction for each submap, we construct two histograms encoding the distribution of circular arc descriptors. In this article we use K-D tree to save the descriptors. Assuming a submap containing arc descriptors with radius range $R\in [R_{min},R_{max}]$ and span angle $A\in [A_{min},A_{max}]$, to obtain a $b_{n}$ bins histogram, we divide the radius into sub-intervals of size $\Delta_{R},\;\Delta_{A}$ as in Equation (18). Each circular arc descriptor falls into a corresponding bin according the radius *R* and angle span *A*, as shown in Equation (19).

(18) $$\begin{array}{cc} \Delta_{R} & =\left(R_{max}-R_{min}\right)/b_{n}\\ \Delta_{A} & =\left(A_{max}-A_{min}\right)/b_{n} \end{array}$$

(19) $$\begin{array}{c} \left(R_{min}+k*\Delta_{R}\right)<R<\left(R_{min}+\left(k+1\right)*\Delta_{R}\right)\\ \left(A_{min}+k*\Delta_{A}\right)<A<\left(A_{min}+\left(k+1\right)*\Delta_{A}\right) \end{array}$$

**Figure 7:**
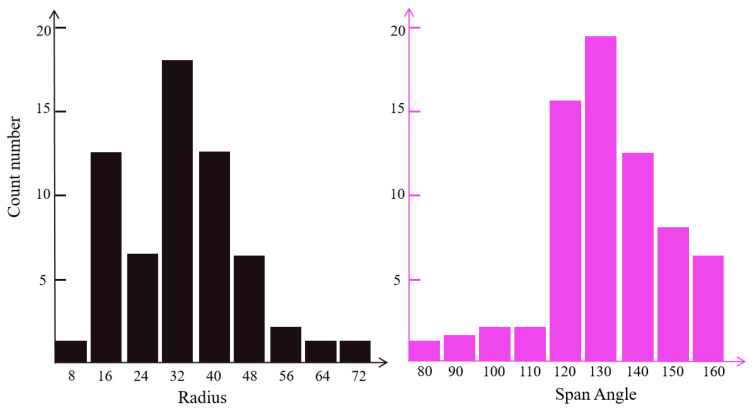
Illustration of histograms based on circular arc descriptors.

The primary advantage lies in its compact representation of global information. Particularly for large-scale environments with complex features, it avoids the redundant feature storage and retrieval inherent in traditional KD-tree approaches. In contrast, histograms demonstrate in Figure 7 superior scalability and adaptability, maintaining O(b) (b is the fixed number of histogram bins) time complexity whether through inserting new submaps or adding descriptors. The second advantage stems from the structural design of our arc descriptor. This architecture enables extraction of simplified yet discriminative features from the environment through quantized circle parameterization.

### 3.4. Loop Detection

#### 3.4.1. Candidate Selection

For the current submap, we first extract VCC descriptors and construct their corresponding histograms based on the above extraction and data storage theory. Then, according to the submap’s position, we retrieve multiple adjacent (spatial distance within $d_{l}$ m) historical submaps as candidates. Specifically, we compare the current submap’s histogram with each candidate through pairwise matching, using Equation (20), and record the results. We select nine as the number of histogram bins ($b_{n}$ = 9), chosen to balance matching accuracy and computational efficiency. After evaluating all candidate submaps, we save the top K matches with highest scores along with their associated VCC descriptors.

(20) $$\begin{array}{c} M_{r}=\sum_{i=0}^{b_{n}}\frac{Qr_{i}}{Nr_{i}}\\ M_{a}=\sum_{i=0}^{b_{n}}\frac{Qa_{i}}{Na_{i}}\\ M=\frac{M_{r}}{b_{n}}\cdot \lambda +\frac{M_{a}}{b_{n}}\cdot \left(1-\lambda \right) \end{array}$$

Here, $M_{r}$ represents the matching result for the radius dimension, where $Nr_{i}$ denotes the count in bin *i* of the radius histogram, while $Na_{i}$ relates to the count in the angle span histogram. $Qr_{i}$ and $Qa_{i}$ denote the angular and radial similarity of descriptors, respectively, between the current and historical submaps within a given statistical bin. Similarly, $M_{a}$ corresponds to the matching result for the angle span dimension, computed through an equivalent process. *M* is the combined matching result, and the parameter λ is a weighting coefficient, with a valid range of [0, 1], which determines the dimensional preference of the matching results. A descriptor pair is considered successfully matched when their similarity scores exceed threshold $M_{\delta }$. We then systematically evaluate all candidate submaps by comparing their histograms against the query histogram. Ultimately, the submap ID achieving the highest matching score is retained for subsequent fine-grained alignment.

#### 3.4.2. Candidate Verification

Following submap-level matching, we perform fine-grained validation on candidate submaps to eliminate globally similar but locally inconsistent matches. Specifically, we first search for arcs with similar shapes, specifically those with a matching radius and span angle, from both the current and matched historical submap. Subsequently, we rigorously evaluate their local geometric attributes, vertical feature types, and associated elevation profiles. This discriminative validation process effectively eliminates spurious correspondences, yielding a robust set of matched circle pairs.

A loop closure is identified when the number of matched VCC pairs exceeds the threshold $M_{\delta }$ (0.3 for this article). Before computing the spatial relationship between descriptors, we first verify the consistency of vertical feature types and their corresponding heights. Then the relative transformation between the current and historical submap is computed via Singular Value Decomposition (SVD) using vertex correspondences from multiple VCC pairs in both submaps.

(21) $$\begin{array}{c} c_{p}=\sum_{i=0}^{3n}\left(p_{i}\right)/\left(3*n\right),\;c_{q}=\sum_{i=0}^{3n}\left(q_{i}\right)/\left(3*n\right)\\ H=\sum_{i=0}^{3n}\left(p_{i}-c_{p}\right)\left(q_{i}-c_{q}\right)\\ H=USV^{T}\\ R=UV^{T}\\ t=c_{q}-R*c_{p} \end{array}$$

The transformation relationship T=(R,t) between the two submaps can be obtained from Equation (21), where $c_{p}$ represents the centroid of VCC vertices in the historical submap, while $c_{q}$ represents the centroid of current VCC vertices. *n* denotes the number of successfully matched VCC descriptors.

Through this verification step, we significantly reduce the probability of false loop closure detection, thereby enhancing system accuracy. After determining the transformation relationship between the current and historical submaps, we further employ the Iterative Closest Point (ICP) [41] algorithm to refine the matching and minimize alignment errors. Through the loop detection process, we identify the historical submap with maximum overlap to the query submap and compute their relative spatial transformation. The transformed VCC descriptors are then updated in the corresponding historical submap.

### 3.5. Parameter Analysis

The parameters employed in the implementation of our proposed VCC framework are systematically cataloged in Table 3. The table provides formal definitions for each parameter: specifically, parameter $C_{n}$ relates to the number of the vertical features used; this parameter directly affects the number of extractable circles, thereby significantly influencing the computational efficiency of the algorithm. $s_{r}$ denotes the radial dimension of the feature search neighborhood, and $n_{a}$ indicates the number of consecutive scan acquisitions aggregated to construct a submap. Lower-resolution LiDAR devices require larger values for the parameter $n_{a}$. Particular attention must be accorded to the calibration of parameters $A_{max}$ and $R_{max}$, as excessively large $A_{max}$ indicates near-collinear feature points, resulting in unreliable spatial relationships, while values that are too small lead to an insufficient number of descriptors and compromised recall. Similarly, an oversized $R_{max}$ introduces redundant descriptors, thereby increasing computational load. $A_{max}$ and $R_{max}$ exert fundamental influence on the geometric properties of the circular arc descriptors, consequently determining both the structural integrity of the VCC representation and the efficacy of candidate submap selection. This relationship directly governs the critical trade-off between recall performance and computational efficiency. $M_{\delta }$ and $d_{l}$ have a similar ratio and search distance to recall and precision; large values of $M_{\delta }$ compromise recall, while insufficiently small values of $d_{l}$ degrade precision. Notably, an identical parameter configuration is maintained across all experimental datasets, while supplementary parameters remain invariant. This methodological consistency substantiates the robustness and environmental adaptability of the proposed algorithm.

## 4. Experiments and Evaluations

### 4.1. Experimental Environment

To validate the effectiveness of our proposed algorithm, we conduct comprehensive experiments on public KITTI [42], NCLT [43], and Mulran datasets [44] to evaluate the performance of our descriptor extraction and loop closure detection methods. The KITTI dataset is collected at 10 Hz using one 64-line spinning LiDAR mounted on the top of moving car in the urban environment. The NCLT dataset, collected at 10 Hz based on one 32-line LiDAR, offers a large-scale, long-term autonomy dataset collected over 15 months on the University of Michigan’s North Campus. The Mulran dataset, collected based on one 64-beam LiDAR, offers urban site datasets with reverse revisits through multi-session and month–evaluation time gaps. To evaluate our algorithm, we select four KITTI sequences (KITTI-00, KITTI-02, KITTI-05, KITTI-08), the four NCLT datasets NCLT-01–NCLT-04 (NCLT2012-05-26, NCLT2012-08-20, NCLT2012-11-17, NCLT2013-04-05), and the four Mulran datasets Mulran-01–Mulran-04 (KAIST01, DCC01, Riverside01, Sejong01). For data processing, we use a computing box equipped with an AMD Ryzen 7940HS processor and 16 GB RAM. This processor is based on the ×86 architecture and is one 16-core CPU with a base frequency of 2.4 GHz.

We compared the proposed algorithm with four existing methods: Scan-Context, M2DP, Scan-Context++ [25], and NDT. Among these, M2DP and NDT can accept submaps as input. Therefore, we merged consecutive $n_{a}$ LiDAR frames with poses to construct submaps as input for these algorithms. Specifically, for the KITTI and Mulran datasets, which use 64-beam LiDAR, we only accumulated two consecutive LiDAR frames as input. For the NCLT and complex urban dataset, we generated submaps by accumulating ten consecutive LiDAR frames. These submaps were then fed into Scan-Context, M2DP, NDT, Scan-Context++, and our proposed VCC algorithm for evaluation. To assess the loop detection performance of the evaluated algorithms, we conducted temporal-based candidate retrieval by selecting historical submaps captured without the two-minute interval of the query submap’s timestamp. All comparative experiments were executed on an identical computing platform.

### 4.2. Qualitative Evaluation of Precision and Recall

In this experiment, we evaluated the qualitative performance (precision and recall) of our proposed method. We comprehensively evaluated the loop closure detection performance of all compared algorithms using precision–recall curves. Specifically, we adjusted the similarity threshold $M_{\delta }$ and $d_{l}$ (check distance) during the candidate selection process to validate the corresponding precision–recall curves at different values. For the other compared methods, we similarly tuned their respective parameters and plotted the corresponding precision–recall curves.

**Figure 8:**
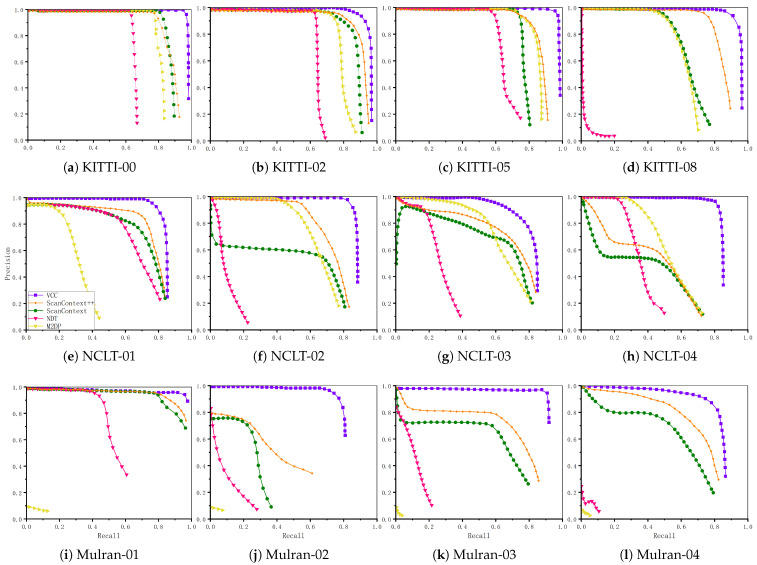
Comparison of precision–recall curves across different methods. Each subplot corresponds to one of the 12 distinct experimental scenarios.

Figure 8 presents the precision–recall curves of all evaluated methods across 12 benchmark datasets. The results demonstrate that our proposed method consistently outperforms the other three baseline approaches in all experimental scenarios. The Scan-Context method performs well in structured environments but exhibits significant performance degradation in unstructured regions. This decline is primarily attributed to Scan-Context’s reliance on globally consistent structural patterns, which become less discriminative when environmental regularity diminishes. The M2DP and NDT methods demonstrate acceptable performance in short-range datasets, but their effectiveness significantly declines in long-distance scenarios. Compared to Scan-Context, the Scan-Context++ descriptor is an extension of Scan-Context, containing richer information, and therefore demonstrates higher accuracy in recall rate. As can be seen, our VCC method sustains 90% precision at 80% recall across all evaluated datasets. Conversely, Scan-Context drops below 70% precision at the same recall level on the challenging NCLT and Mulran datasets, and both NDT and M2DP suffer from more severe declines in both recall and precision. In summary, our proposed VCC method maintains high precision while achieving superior recall rates in complex, long-range, unstructured environments.

**Figure 9:**
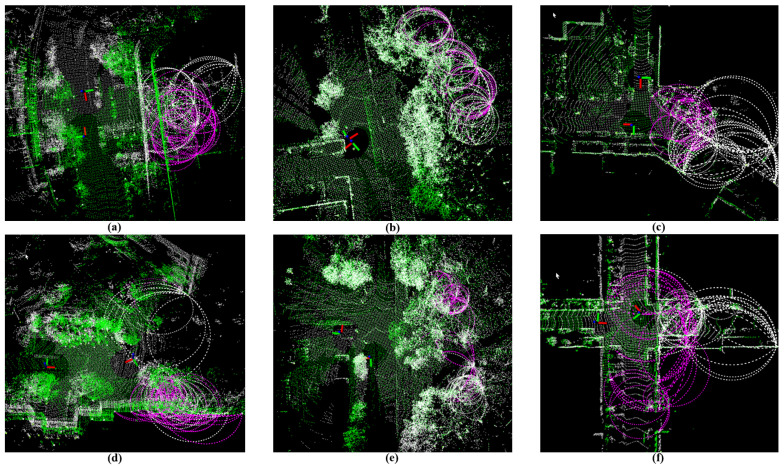
Successful loop closure detection in challenging scenarios from the datasets. Point cloud in white is historical submap ($M_{\delta }=0.5,d_{l}=50$ m). White arcs are descriptors extracted from the historical submap, while the green cloud and magenta arcs represent the current submap and the corresponding descriptors. Different scenes (**a**) large angle bias in KITTI-00; (**b**) large angle bias in NCLT-02; (**c**) non-overlapping regions in KITTI-08; (**d**) large spacing scene in KITTI-08; (**e**) large spacing scene in Mulran-04; (**f**) non-overlapping regions in Mulran-02.

To further validate the robustness of our proposed VCC method, we selected several challenging scenarios where other methods struggled to achieve loop closure matching due to limitations in pose estimation. These scenarios were extracted from the dataset, as illustrated in Figure 9.

Figure 9 presents several challenging loop closure detection scenarios. The results demonstrate the robustness of our proposed VCC method under various difficult conditions. To better demonstrate the effectiveness of our algorithm, Figure 9a,d present the raw data of the loop closures identified by the algorithm without performing registration; significant deviation between the origin data can be observed. The remaining illustrate cases where point cloud registration is fully completed. The point cloud of the historical submap is shown in white, while the current submap is shown in green. KITTI-00 (a) (delta angle 175°) and NCLT-02 (b) (delta angle 175°) present a scenario of large perceptual differences between the historical and current scene when traversing the same location in opposite directions. VCC successfully handles significant viewpoint changes through its rotation-invariant descriptor design. KITTI-08 (d) (distance 23 m, delta angle 99.5°) and Mulran-04 (e) (distance 29 m, delta angle 151.3°) demonstrate that our VCC method effectively handles loop closure detection even when substantial deviations exist between historical and current poses. KITTI-08 (c) and Mulran-02 (f) illustrate scenarios with non-overlapping regions between frames. Our VCC method can identify valid loop closure descriptors despite these challenges, which arise from isolated data segments between current environmental information and queried environments.

### 4.3. Quantitative Analysis on Pose Estimation Accuracy

**Figure 10:**
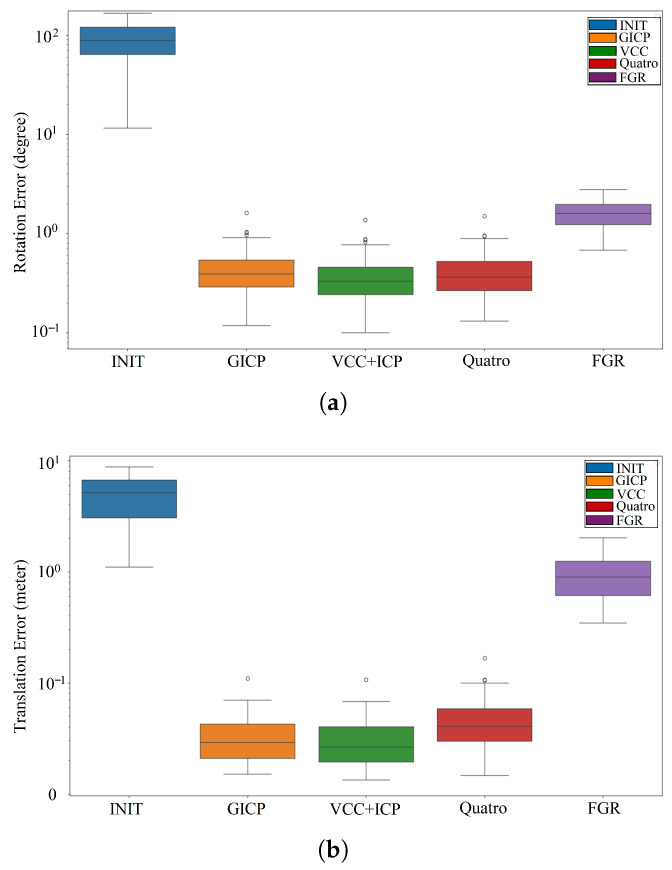
Position error evaluation on dataset KITTI00 under different translational and rotational error settings. (**a**) Translation error between different methods. (**b**) Rotation error between different methods.

In this section, we evaluate the pose estimation accuracy of the proposed algorithm. Based on our provided coarse six-DoF relative transformation between the current frame and the loop closure frame, a subsequent refinement step using the Iterative Closest Point (ICP) algorithm is implemented. Some other loop closure methods cannot provide complete six-DoF pose estimation results (Scan-Context, M2DP). Here, we compare our approach (VCC+ICP) with GICP [45], Quatro [46], and FGR [47]. The results on KITTI00 sequences are reported in Figure 10. Our proposed VCC+ICP method demonstrates superior performance, showing minimal deviation compared to other methods. Furthermore, our model holds particular advantages in urban road environments and indoor scenes with structured vertical features. Even when the source and target clouds are in completely opposite directions, our method still maintains low rotational error Figure 10a. To test the robustness of pose estimation under different translation errors, we perturb the initial relative pose for each loop pair at varying scales, applying perturbations within a certain range (translational perturbation up to 8 m). Figure 10b shows the translation errors for different methods. VCC+ICP achieves better accuracy comparable to other methods. This is because VCC provides a good initial value for ICP fine registration through coarse matching, and by superimposing rotational offsets, it can simulate uneven ground conditions. Consequently, the results also confirm that our algorithm can handle non-planar motion.

To validate the universal applicability of the proposed algorithm across different radar systems, we conducted additional experiments using two test datasets, KITTI-05 (64-beam) and NCLT-02 (32-beam), with different initial offset values. The experimental results (Te—translation error(m), Re—rotation error(deg)) in Table 4 demonstrate that the VCC algorithm exhibits remarkable robustness against both rotational and translational perturbations, maintaining consistent performance under both small and large initial pose disturbances. Since the algorithm takes voxelized point cloud data as input, the difference beam LiDAR data does not affect the algorithm’s performance results. This stability stems from its ability to extract complete features within the local coordinate system of the point cloud, thereby remaining unaffected by initial pose perturbations. In contrast, methods such as GICP, Quatro, and FGR, which rely on good initial pose estimation to achieve convergence, experience significant accuracy degradation when subjected to large pose disturbances.

### 4.4. Time Consumption

Vertical features can be used to construct the VCC descriptors, which in turn enable the spatial localization of objects based on these descriptors. The accuracy and completeness of the VCC are directly related to the integrity of environmental description, which is critical information for loop closure verification in SLAM (Simultaneous Localization and Mapping). Therefore, to evaluate the efficiency of our method, we conduct an experiment to compute the time efficiency of the proposed approach, Scan-Context, M2DP, NDT, and Scan-Context++ across 12 diverse scenarios. The time efficiency experiment will focus on the average time of the descriptor construction procedure and loop detection procedure and the total submap adjustment time. All experiments are conducted under an identical system equipped with an AMD Ryzen 7940HS processor and 16 GB RAM, running the ubuntu x64 operating system. The time costs of all methods are shown in Table 5.

The results show that, across all scenarios, the proposed algorithm achieves the lowest total time consumption for each submap. In highly structured environments (KITTI), the Scan-Context algorithm exhibits the highest descriptor extraction efficiency, while the VCC method follows closely in descriptor extraction performance. However, Scan-Context’s loop closure query is less efficient compared to VCC, primarily because VCC utilizes a histogram-based pre-verification approach to rapidly filter valid candidate submaps for matching. The NDT method incurs the highest computational cost due to its point cloud matching process for large-scale submaps, and this issue is exacerbated in datasets with dynamic elements (Mulran). The M2DP method demonstrates significant advantages in loop closure matching efficiency, but its descriptive capability becomes ambiguous and unreliable when facing unstructured outdoor scenes.

### 4.5. Ablation Study

This section presents an ablation study evaluating the proposed algorithm. The experiments herein investigated the impact of the proposed method on matching precision, accuracy (translation error, rotation error), and computational efficiency. We selected three datasets—KITTI-00, NCLT-01, and mulran-01—for the ablation validation. Under consistent parameter settings, specifically using identical keyframes from the same datasets, we evaluated the performance of three configurations: VCC+ICP, VCC alone, and ICP alone. To evaluate matching results, deviations of varying magnitudes were applied to the ground-truth pose (translation/rotation) of selected loop closure frames. Matching accuracy was measured by the difference between estimated results and the introduced deviations. A match was considered successful when the matching error was less than 50% of the preset error. As summarized in Table 6, the VCC+ICP combination achieved significantly higher accuracy than either VCC or ICP individually, albeit with a modest increase in computational time. The improvement in both accuracy and matching efficiency was attributed to the high-quality initial pose provided by the VCC descriptor. These findings underscore the importance of the VCC descriptor, even with large deviations.

**Figure 11:**
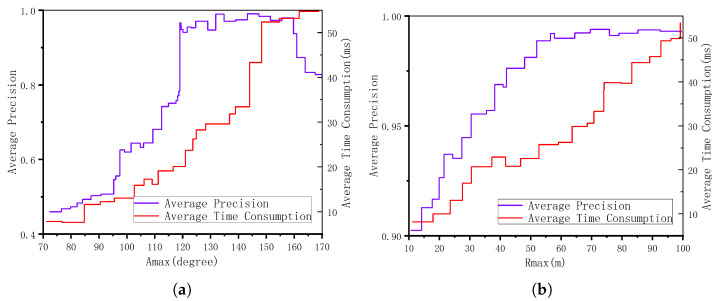
Ablation study: Analyzing the effect of parameters $A_{max}$ and $R_{max}$ on precision and computation time. Results are presented on dataset kitti-02: (**a**) Impact of parameter $A_{max}$ on the loop closure detection performance. (**b**) Impact of parameter $R_{max}$ on the loop closure detection performance.

Another ablation study was conducted to evaluate the influence of parameter $A_{max}$ on loop closure detection performance. The value of $A_{max}$ was systematically varied within the loop detection pipeline while the $R_{max}$ was fixed, with experiments performed on the KITTI 02 dataset. Figure 11 illustrates the corresponding average precision and computational time. The results indicate that increasing $A_{max}$ enhances the number of descriptors and submap matches, which improves precision at the expense of greater computational load. However, beyond $A_{max}=160$, no further precision gain is observed, as angular artifacts begin to generate anomalous descriptors. Therefore, to balance precision and efficiency, $A_{max}=140$ was selected. A similar ablation analysis was performed for parameter $R_{max}$. Increasing $R_{max}$ incorporated more descriptors into the matching process, improving precision while increasing computational cost. Beyond $R_{max}=70$, no significant improvement was achieved; thus $R_{max}=70$ was chosen for practical implementation.

## 5. Conclusions

This paper proposes the VCC descriptor, a novel loop closure detection method leveraging the spatial distribution of circular arcs. By utilizing a set of descriptors to represent global environmental features and further verifying them with local height attributes, the method enhances the system’s capability for multi-scale place recognition. We establish rules for ordering the points constituting the arcs, ensuring the rotation and translation invariance of the arc descriptors across varying viewpoints. This construction approach not only enables accurate and efficient place recognition but also guarantees the robustness of the system. Experimental results demonstrate that the VCC method outperforms existing approaches in both loop closure detection capability and computational efficiency across diverse environments and LiDAR sensors. Notably, it maintains high precision and strong adaptability even in challenging scenarios such as reverse revisits, significant pose variations, and low-overlap regions.

While the proposed algorithm performs well, it still exhibits limitations in certain scenarios. For instance, in environments with high geometric similarity or symmetrical layouts, the method remains prone to erroneous place recognition. Additionally, highly uniform environmental features or significant feature occlusion can lead to failures in environment identification.

## Figures and Tables

**Table 1:** Comparison of key LiDAR global descriptors.

Method	Core Principle	Feature Representation	Invariance Implementation	Matching Complexity	Key Limitations
M2DP	Multi-view 2D projection	1D Feature Vector	PCA-based axis alignment	O(n)	Loss of spatial topology; sensitive to noise.
NDT	Normal Distribution	Gaussian PDF Grids	Initial pose-dependent	Iterative	Designed for local registration; local loop closure retrieval.
Scan Context	Polar Height Projection	2D Matrix (Rings × Sectors)	Brute-force circular shifting	O(nlog(n))	High computational cost for rotation alignment; sensitive to lateral translation.
BTC	Triangle Binary	Local Triangle Binary Bitstream	Local structural encoding	$O(N_{tri})$	Sensitive to point cloud sparsity; triangle shape varies with viewpoint.
STD	Stable Triangle	Local Triangle Geometry	Euclidean invariants (sides/angles)	$O(N_{tri})$	Sensitivity to keypoint extraction noise; susceptible to combinatorial explosion.
VCC (Ours)	Vertical Circular	Geometric Invariants (R,θ)	Intrinsic SE(2) invariance	$O(N_{c})$	Requires vertical features; stable with sparse data.

**Table 2:** Grid classification based on curvature.

Grid Classes	Angle	Direction
Corner	$A_{i,j}$ > Amin and $A_{i,j}$ < Amax	$V_{l}$ and $V_{r}$
Planar	$A_{i,j}$ > Amax	$V_{l}$ and $V_{r}$
Nonsense	$A_{i,j}$ < Amin	$V_{l}$ and $V_{r}$
No label	-	$!V_{l}$ or $!V_{r}$

**Table 3:** Parameters used for the experiments.

Parameter	Value	Description
$C_{n}$	20	Closest vertical feature number
$A_{max}$, $R_{max}$	140°, 70 m	Max angle span, max radius
$s_{r}$	7	Neighbor grid search radius
$n_{a}$	2–15	Accumulation number of scans
$M_{\delta }$	0.3–0.7	Ratio of similar descriptors
$d_{l}$	30 m–70 m	Distance to candidate submap

**Table 4:** Comparison of pose estimation with the state-of-the-art methods on KITTI-07 and NCLT-02 with different initial offset.

Dataset	Method	0~2 (m) 0~10 (°)	4~6 (m) 30~40 (°)	8~10 (m) 60~80 (°)
		Te/Re	Te/Re	Te/Re
KITTI-07	VCC+ICP	0.067/0.152	0.069/0.155	0.067/0.155
	GICP	0.102/0.278	0.115/0.295	0.175/0.317
	Quatro	0.052/0.215	0.103/0.228	0.424/0.325
	FGR	0.055/0.218	0.096/0.298	1.542/1.563
NCLT-02	VCC+ICP	0.089/0.229	0.093/0.231	0.094/0.232
	GICP	0.235/0.395	0.314/0.463	0.552/0.573
	Quatro	0.085/0.263	0.146/0.317	0.578/0.397
	FGR	0.083/0.258	0.233/0.325	1.775/1.596

**Table 5:** Time consumption of each method among different experimental environments.

Scene Feature	Dataset/ Length [km]	Average Points		Descriptor Extraction [ms]/Loop Detection [ms]/Total Time [ms]			
			VCC	ScanContext	M2DP	NDT	ScanContext++
mixed scene	NCLT-01/ 6.3	155,607	19.33/4.72/24.05	23.83/23.25/47.08	35.49/2.52/38.01	85.23/550.31/635.54	35.74/22.64/58.38
	NCLT-02/6.0	168,739	19.52/5.57/25.09	24.64/23.43/48.07	49.36/3.74/53.10	84.51/544.75/629.26	36.33/21.41/57.74
	NCLT-03/5.7	159,712	20.03/4.32/24.35	23.63/23.83/47.46	39.54/2.18/41.72	80.42/470.16/550.58	36.13/23.13/59.26
	NCLT-04/4.5	148,207	20.13/5.72/25.85	24.05/23.64/47.69	58.17/1.75/59.92	130.12/510.77/655.89	34.95/22.44/57.39
structure urban	KITTI-00 /3.72	121,134	12.05/3.93/15.98	11.45/24.34/35.79	39.64/1.01/40.65	46.35/210.15/256.50	22.23/22.83/45.06
	KITTI-02/5.06	123,092	15.34/5.72/21.06	10.85/23.87/34.72	43.55/2.51/46.06	41.56/194.41/235.97	21.51/21.93/43.43
	KITTI-05/2.21	122,235	13.53/3.12/16.65	11.05/25.17/36.22	41.79/1.82/43.61	39.45/157.81/197.26	20.75/23.14/43.89
	KITTI-08/2.45	121,494	14.12/5.62/19.74	12.57/23.36/35.93	52.37/2.06/54.43	49.73/224.51/274.24	22.23/24.27/46.50
reverse revisit, dynamic object	Mulran-01/4.9	141,860	17.75/1.93/19.68	21.31/23.56/44.87	73.22/2.52/75.74	169.15/176.63/345.78	31.92/21.42/53.34
	Mulran-02/6.1	145,394	18.12/1.38/19.50	22.45/22.77/45.22	70.15/1.33/71.48	120.31/193.32/313.63	32.74/21.87/54.61
	Mulran-03/6.8	155,252	18.64/1.35/19.99	23.14/23.41/46.55	79.34/1.15/80.49	185.80/276.15/461.95	33.05/23.41/56.46
	Mulran-04/23.4	157,314	20.52/4.67/25.19	24.07/22.28/46.35	91.17/2.74/99.91	312.43/423.34/735.77	32.87/23.12/54.99

**Table 6:** Ablation study: Comparison of loop matching results. Large deviation (5 m–9 m, 70°–90°) and small deviation (0.1 m–2 m, 1°–10°).

Dataset	Deviations	Precision (%)/Translation Error (m)/ Rotation Error (deg)/Time Consumed (ms)		
		VCC+ICP	VCC	ICP
KITTI-00	Small	100/0.049 0.161100/83	100/0.085 0.233100/23	95.31100/0.115 0.321100/113
	Large	100/0.047 0.157100/93	100/0.095 0.233100/25	45.28100/0.255 0.461100/1094
NCLT-01	Small	100/0.039 0.154100/125	100/0.083 0.230100/30	91.68100/0.175 0.397100/145
	Large	100/0.042 0.163100/109	100/0.093 0.247100/32	31.54100/0.315 0.564100/1309
Mulran-01	Small	99.7100/0.055 0.167100/98	99.7100/0.105 0.245100/27	66.4100/0.216 0.435100/127
	Large	99.7100/0.057 0.164100/101	99.7100/0.105 0.254100/29	29.02100/0.335 0.523100/1215

## Data Availability

The original data presented in the study are openly available in Data_test at https://pan.baidu.com/s/1Y1NzdoV5uoLk-niOXlz_5g?pwd=5577[5577] (accessed on 6 February 2026).
